# Does the Incredible Years Teacher Classroom Management Training programme have positive effects for young children exhibiting severe externalizing problems in school?: a quasi-experimental pre-post study

**DOI:** 10.1186/s12888-016-1077-1

**Published:** 2016-10-26

**Authors:** Bente Kirkhaug, May Britt Drugli, Bjørn Helge Handegård, Stian Lydersen, Merethe Åsheim, Sturla Fossum

**Affiliations:** 1 The Regional Centre for Child and Youth Mental Health and Child Welfare – Central Norway, Norwegian University of Science and Technology (NTNU), Norway, Klostergata 46, Trondheim, 7030 Norway; 2 Centre of the Study of Educational Practice (SePU), Hedmark University College, Hedmark, Norway; 3 The Regional Centre for Child and Youth Mental Health and Child Welfare – North Norway. Faculty of Health Sciences at UiT, Artic University of Norway, Trondheim, Norway

**Keywords:** Severe externalizing problems, School universal intervention, IY TCM, Young school children, Teacher assessment, Norway

## Abstract

**Background:**

Young children exhibiting severe externalizing problems in school are at risk of developing several poor outcomes. School-based intervention programs have been found to be effective for students with different problems, including those with behavioral problems, emotional distress, or social problems. The present study investigated whether the IY-TCM programme, as a universal stand-alone school intervention programme, reduced severe child externalizing problems as reported by the teacher, and evaluated if these children improved their social competence, internalizing problems, academic performances and student- teacher relationship as a result of the IY TCM training.

**Methods:**

A quasi-experimental pre-post study was conducted, including 21 intervention schools and 22 control schools. Children in 1^st^ – 3^rd^ grade (age 6–8 years) assessed by their teacher as having severe externalizing problems on the Sutter–Eyberg Student Behavior Inventory-Revised (SESBI-R) total Intensity score, were included in the study, *N* = 83 (65 boys and 18 girls). Treatment effects were evaluated using 3- level linear mixed models analysis.

**Results:**

In our study we found no differences in change between the two conditions from baseline to follow-up in externalizing problems, social skills, internalizing problems and closeness with teacher. The intervention condition did however show advantageous development in terms of student-teacher conflicts and increased academic performances.

**Conclusion:**

The IY Teacher Classroom Management program is not sufficient being a stand-alone universal program in a Norwegian primary school setting, for students with severe externalizing problems. However; some important secondary findings were found. Still, young school children with severe externalizing problems are in need of more comprehensive and tailored interventions.

## Background

Severe externalizing problems often grow from early minor problems into more serious problems because of negative interactions over time between the child and the environment [1]. School plays a crucial role in this process. Here externalizing problems are defined as severe attention problems, rule-breaking and aggressive behavior problems [2]. Children with externalizing problems in school are at risk of poor outcomes, including inferior school performance, poor personal adjustment, low social competence, peer difficulties, conflict with teachers, negative school adjustment, school drop-out, and future criminal behavior and unemployment [3–7]. It is estimated that 5–10 % of all Western children exhibit severe externalizing problems [8]. In Norway, these numbers include 2–3 % of children aged 4–12 and are among the lowest in the world [9–11].

Teachers identify children as having severe externalizing problems when the children demonstrate the following behaviors frequently and with high intensity: inattentiveness, impulsiveness, fails to finish tasks, poor academic work, brakes rules, lie or cheat, run away from the classroom, feels no guilt, are mean, argue, fight, attacks, and so on [2, 12, 13]. For children with severe externalizing problems, associated impairments is the rule rather than the exception [14]. ADHD, internalizing problems, academic failure and a lack of social competence are the most common impairments that accompany child externalizing problems [3, 10, 15–17]. Further. associated impairments in the child is related to higher risk of negative psychosocial outcomes in adolescence and early adulthood [16]. Negative behavior elicits considerable attention from teachers and can overshadow co-occurring problems such as anxiety or depression [14]. Children who exhibit severe externalizing problems in school often receive more negative feedback from teachers than their more behaviorally competent peers do. To a large extent, children’s academic problems can be attributed to externalizing problems and can leave them ill prepared to learn, thereby decreasing their cognitive abilities [18, 19]. However, externalizing and academic achievement are reciprocally related [20]. Severe externalizing problems in school are often time consuming and detract from valuable teaching and learning time. Therefore, decreasing the incidence of externalizing problems in the classroom can have a substantially positive impact on school achievement for all children, particularly children at risk [21].

Effective classroom management and positive behavioral motivation may prevent externalizing problems and improve social as well as academic functioning among children [22–24]. In contrast, poor classroom management is associated with an increased risk of social and behavioral problems [25], less academic instruction and a negative learning environment [26]. Numerous studies emphasize the importance of positive teacher classroom management strategies, such as establishing firm and clear rules, being polite, giving emotional support, using praise, motivating child learning, giving children greater responsibility and choices, performing a high level of monitoring, enhancing academic achievement and school readiness, being flexible when using rewards and sanctions, and responding to disruptive behavior in adequate ways [4, 27–32]. When a teacher succeeds in managing the classroom, the students better understand how to behave, and the majority of classroom time can be spent in learning activities [33].

Teaching involves continuous interactions between the instructor and students. Individual differences in students require support and attention from skilled teachers who play an important role in helping children to make the most of their school achievements [34]. Externalizing behavior and the quality of the student- teacher relationship can be bi-directional [20, 35, 36]. Positive teacher-student relationships have been found to play a significant role in preventing externalizing problems [37]. For students at risk of negative development, positive student–teacher relationships serve as a resource to prevent school failure, whereas conflict and disconnection between teachers and students may enhance that risk [38]. Studies have found that students whose relationships with teachers are characterized by greater closeness and less conflict display lower levels of aggression and fewer externalizing problems [36, 37, 39]. These types of positive relationships with teachers can act as an compensatory resource for these children, but few classroom management intervention studies have focused on the effects of teacher-student relationships [36].

Several interventions aim to improve classroom behavior by targeting specific children with externalizing problems [23, 27]. However, working directly with teachers to improve the classroom and school social environment might be beneficial for improving behavior as well as academic functioning among children. Positive side effects of such approaches include improvements to the quality of classroom management and the school climate for both students and teachers. School-based intervention programs have been found to be equally effective for students with various problems, including behavioral problems, emotional distress, or social problems [27, 40, 41].

Significant long- and short-term effects on behavioral change within both universal and indicated programs in school have been found [27, 40, 42–44]. The effect sizes of universal and indicated preventive programs of behavioral change in school-based intervention programs typically range from small to moderate, with indicated programs often showing somewhat lager effects than universal programs [40, 42]. Research also shows that the youngest children gain more from intervention programs than older children do [27, 28, 40]. In contrast, studies show that when no intervention is used, very small changes or even negative changes in children’s behavior are reported [45].

In the present study, an evaluation of The Incredible Years (IY) Teacher Classroom Management program (TCM) was conducted in Norwegian schools. The IY TCM is part of three interconnected and complementary IY training programs comprising parent, child, and teacher training [46]. The IY TCM is a universal preventive school intervention to strengthen teachers’ classroom management strategies. Teachers can apply these strategies to all children in the class as well as to those with severe externalizing problems [47]. The IY TCM is designed to reduce risk factors associated with classroom management practices, early onset externalizing problems, and emotional difficulties and emphasizes how teachers can effectively collaborate with parents and schools [47]. Furthermore, the IY TCM program is developed to help teachers manage disruptive and problem behavior in the classroom and to promote school readiness and children’s prosocial behavior [22].

To date, results from evaluations in the United States of the IY TCM intervention program have consistently supported the intervention. Studies have documented an increase in teachers’ use of effective classroom management strategies, such as using more praise and being more nurturing, consistent, and confident, and a decrease in child externalizing problems [41, 48–50]. Additionally, children in classrooms with teachers who received IY TCM in combination with parent training show less aggression toward peers and more cooperation with teachers [4, 51].

The IY TCM program has shown promising results in the U.S.; however, these results are consistently found in combination with parent training and/or child training. Few studies outside the U.S. have examined the effect of the IY TCM as a stand-alone intervention. Hutchings et al. [44] is the only previous study to our knowledge that has evaluated the IY TCM as a stand-alone intervention in elementary schools outside the U.S. In their study, Hutchings et al. [44] included a group of children with severe externalizing problems who were rated as above the point of clinical concern; however, with 18 children the sample size was fairly small. Significant positive effects on the IY TCM were found within the targeted students, who showed a reduction in negative behavior toward teachers and in off-task behavior [44].

This study aimed to assess whether the IY-TCM program, provided as a universal stand-alone program, reduces severe child externalizing problems as reported by the teacher. Another aim was to evaluate whether these children improved their social competence, internalizing problems and academic performance. A final aim was to explore whether the student-teacher relationship would improve as a result of the IY TCM. In accordance with previous research, we hypothesized that the children in the intervention condition would demonstrate reductions in problem behavior and internalizing problems, increased social competence and academic performance, and an improved student-teacher relationship compared to the control condition.

## Method

### Participants

The IY Norway invited Norwegian municipalities that had previously implemented the IY Parenting Training program, and hence could be trained as group leaders for the school program, to participate in this research study and receive the implementation of the IY TCM in their schools. The recruitment was performed by announcing and informing the appropriate education agencies in the municipalities about the implementation and research study of the IY TCM program. Interested schools applied to IY Norway for participation. The 21 intervention schools that participated in the research study received the implementation of the IY TCM for free. In addition, each participating school and contact teacher received a small financial compensation for the time spent to complete the research questionnaires.

**Fig. 1:**
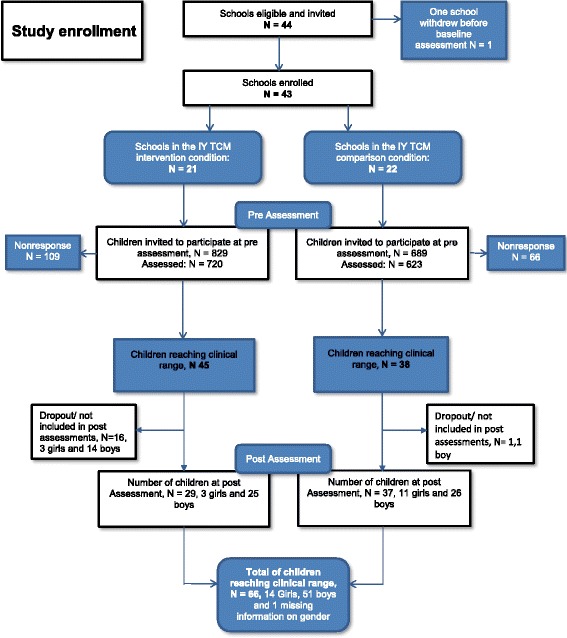
Study Enrollment

Another 22 schools were recruited to the control condition. These schools were from rural and urban municipalities throughout Norway. Schools in the control condition were offered financial compensation for not immediately receiving the intervention and for the teacher’s time spent completing the research questionnaires pre- and post-intervention. The control condition schools were offered training in the IY TCM program a year later. To avoid program contamination, an inclusion criterion for the entire sample was that none of the schools were currently attending or had attended any other evidence-based school behavior intervention programs for the last year. The schools had to fill out a brief questionnaire and only schools who answered no were considered eligible and hence invited to participate in the study. All school leaders responded negatively to this question, resulting in 44 enrolled schools that were divided into an intervention or a control condition (see Fig. 1 for study enrollment). Parents who did not speak or understand Norwegian were excluded. Data were collected only on children whose parents had given consent, but all children in the intervention classrooms received the classroom intervention.

To reduce each teacher’s burden and dependency on the data in completing the assessments of their students, seven students per class were randomly selected for pre assessments on different measurements. For example, in a class of 21 students, a statistical random number sequence list from 1 – 21 was generated electronically, and the teachers matched the first seven numbers with the children’s alphabetical placement on a class list in a predetermined way. A statistician blind to the characteristics of the classroom or the school conducted the randomization. All children included in this study was screened using the Sutter–Eyberg Student Behavior Inventory-Revised (SESBI-R), and scored equal to or above the 90^th^ percentile on the SESBI-R total intensity score [52]—that is, a score of at least 144, which is equivalent to the clinical range. Out of a total of 1343 children, 83 children (6 %) scored above the cut-off. Of these children, 45 were from schools using IY TCM (38 boys and 7 girls) and 38 children were from control schools (27 boys and 11 girls). Forty-one percent of the children reaching clinical range receive special educational assistance in class. See table 1 for the demographic characteristics of the sample.

**Table 1:** Demographic characteristics of the sample, *N* = 83

Demographic characteristics	IY TCM	Control	Total
Child			
N Grade^a^	45 (100 %)	38 (100 %)	83 (100 %)
1^st^ grade	14 (31,1 %)	14 (36,8 %)	28 (33,7 %)
2^nd^ grade	11 (24,4 %)	8 (21,1 %)	19 (22,9 %)
3^rd^ grade	20 (44,4 %)	16 (42,1 %)	36 (43,4 %)
Gender^b^			
Boys, *n* (%)	38 (84.4 %)	27 (71.1 %)	65 (78.3 %)
Girls, *n* (%)	7 (15.6 %)	11 (28.9 %)	18 (21.7 %)
Teacher^c^			
N	35 (100 %)	31 (100 %)	66 (100 %)
1^st^ grade	11 (31.4 %)	11 (35.5 %)	22 (33.3 %)
2^nd^ grade	9 (25.7 %)	6 (19.4 %)	15 (22.7 %)
3^rd^ grade	15 (42.9 %)	14 (45.2 %)	29 (44.0 %)
School			
N	16	18	34

Notes: Tests for group differences: ^a^ *p* = .85, ^b^ *p* = .14, ^c^ *p* = .82

All schools completed the IY TCM training, however high attrition on the pre-post assessments was found for the sample, demonstrating a different dropout pattern between the intervention and control conditions. In the intervention condition, a total of 16 individuals dropped out by the post-assessments. Two were related to school dropout, eight were related to teacher dropout, and six were related to student/family dropout. In comparison, 2 individual dropouts were related to the student/family in the control condition. By matching personal information about the teacher’s age, sex, work experience, type of position etc. at both time points, it was found that it was the same teacher who did both pre and post assessments in 95.8 % of the cases. Statistically significant differences were found between the grades, with fewer dropouts in 3^rd^ grade (8 %) compared to 32 % dropouts in 1^st^ and 2^nd^ grade. Equivalent dropout between the conditions was found between genders, students who received special education and those who did not, and the teacher’s familiarity with the student.

### Design

This study had a quasi-experimental pre-post design with intervention and control schools. Continuous enrollment of intervention and control schools was conducted from the fall of 2009 to the fall of 2013, and the final post-assessments (T2) were collected during the spring of 2014.

### Procedures

Prior to the pre-intervention assessment and the first IY TCM training, the study was presented to the school staff. Teachers for students aged 6 to 8 years attending 1^st^ through 3^rd^ grade and staff in after-school services were invited to attend. Information about the project was presented to the intervention and control schools separately. Parents of students in 1^st^ – 3^rd^ grade received written information about the data collection procedures and were asked to consent to their children’s participation. The teachers received the questionnaires 1 to 3 weeks before the first workshop session training in IY TCM and again 1 to 3 weeks after the final workshop. The period between the two assessments typically lasted 8–9 months. Because the IY TCM is a universal preventive program, teachers and after-school staff were trained simultaneously.

### Measures

Externalizing problems were measured with the Sutter–Eyberg Student Behavior Inventory-Revised (SESBI-R), which consists of 38 items used by teachers to evaluate the current frequency and severity of various types of behavior in children aged 2–16 years. The items describe common behavioral problems that are observable by teachers, such as “has temper tantrums”, “pouts”, “acts defiant when told to do something”, “has difficulty staying on task”, “has trouble paying attention” and “fails to finish tasks or projects” [53], which are rated on a seven-point intensity scale: 1 = never, 2–3 = seldom, 4 = sometimes, 5–6 = often and 7 = always [53]. Total scores are summed across all items on the Intensity scale; the total ranges from 38 to 266. A score of 144 or more is equal to the clinical range. The SESBI-R has been found to be a reliable and valid instrument for efficient screening and tracking of the behavior of conduct-disordered children [52, 53]. In this study, we used the SESBI-R Intensity score with Norwegian norms for 3- to 8-year-old children [52]. The internal consistency for the entire sample was measured with Cronbach’s alpha and found to be .97.

Internalizing problems and academic performance were measured using the Teacher Report Form (TRF), a part of the ASEBA (Achenbach System of Empirically Based Assessment) family of instruments [2]. The TRF contains teacher ratings of children’s conduct problems, academic performance and adaptive characteristics [2]. The Internalizing scale comprises the subscales Anxious/Depressed (16 items), Withdrawn/Depressed (8 items), and Somatic Complaints (9 items). The Internalizing scale in this study was used as a measure of a child’s internalizing problems. Teachers were asked to rate the degree of the child’s emotional problems, including items such as “Must be perfect”, “Feels worthless”, “Enjoys little”, “Withdrawn”, “Stomachaches” and “Headaches”, for the previous 2 months on a 0–2 scale (0 = not true as far as you know; 1 = somewhat or sometimes true; 2 = very true or often true). Summed across all items, the Internalizing total score ranges from 0 to 66. Internal consistency for the entire sample based on Cronbach’s alpha for the TRF internalizing subscales in this study was 0.79 (Anxious/Depressed), 0.72 (Withdrawn), and 0.53 (Somatic Complaints). In addition, the academic performance scale was used. The academic performance scale rates the child’s overall and current academic performance. Each teacher was asked to assess and compare with class averages the children in six different academic subjects of the teachers choosing. On a scale from 1 to 5 (1 = Far below grade, 2 = Somewhat below grade, 3 = At grade level, 4 = Somewhat above grade and 5 = Far above grade). Summed across all subjects, a mean score was calculated. Test–retest reliability and validity have been found to be high [2].

Social skills were measured using the Social Skills Rating System (SSRS). The SSRS measures the occurrence and importance of specific social skills, academic competence and behavioral problems as perceived by teachers [54]. The SSRS contains 57 items and offers a broad assessment of a child’s social behavior. In the present study, the 30 items of the Social Skills subscale were utilized, including items such as “makes friends easily”, “controls temper in conflict situations with peers”, “gets along with people who are different” and “follows directions”. The teacher evaluates how often each social skill occurs on a 0–3 scale: 0 = never, 1 = sometimes, 2 = often, 3 = very often. Summed across all items, the scores range from 0 to 90. Both test–retest reliability and the validity of the SSRS have been found to be good [54]. Internal consistency for the entire sample of the SSRS Social Skills Subscale using Cronbach’s alpha in this study was .94.

The student-teacher relationship was measured using the Student-Teacher Relationship Scale, short form (STRS-SF), which is a widely used rating scale to examine teachers’ relationships with their students. In this study, the STRS-SF was adjusted for the Norwegian population [55]. The STRS-SF consists of a 15-item scale used to assess teachers’ perceptions of two features of their relationships with their students: closeness and conflict [56]. The Closeness subscale contains 8 items (scores ranging from 8 to 40) related to the degree of warmth and open communication in the teacher child relationship and includes items such as “I share an affectionate, warm relationship with this child” and “It is easy to be in tune with what this child is feeling”. The Conflict subscale contains 7 items (scores ranging from 7 to 36) measuring the extent to which the teacher-child relationship is characterized by antagonistic, disharmonious interactions, and includes items such as “This child is easy to be in tune with what this child is feeling” and “This child easily becomes angry with me”. Internal consistency for the entire sample using Cronbach’s alpha for STRS-SF in this study was 0.81 for closeness and 0.84 for conflict.

### The IY TCM intervention

The IY Teacher Classroom Management program is a prevention program developed to strengthen teachers’ classroom management strategies and to promote children’s school readiness and prosocial behavior [47]. In this study, classroom teachers and staff in after-school services received the IY TCM intervention to strengthen their skills in effective classroom management. The program includes research-based classroom management strategies that have been associated with increases in children’s social and emotional development, positive teacher-student interactions and decreases in students’ externalizing problems [57].

The IY TCM program includes 6 full-day workshop sessions, led by two experienced and qualified IY TCM group leaders. Most group leaders had a master’s degree in special education, and a few had a minimum of 3–4 years of higher education within health and social studies/a Bachelor degree. All group leaders had several years of work experience. Further, group leader qualifications such as personal suitability, motivation, good relational skills and collegial respect was highly recommended. Group leaders participating in this research study, also had to have delivered the IY TCM training on several prior occasions.

Each workshop session last 6–7 h, and the time period between each workshops is 3 – 4 weeks. Between workshops, the teachers are practicing the new skills they are learning. In addition they receive either verbal or written feedback/guidance from the group leaders on classroom based practice of new skills, in addition to verbal and written assignments between each workshops.

The IY TCM training involves the following themes covered in 6 workshops, with each workshop building on the previous ones: Building Positive Relationships with Students and The Proactive Teacher; Teacher Attention, Coaching, Encouragement & Praise; Motivating Students through Incentives; Decreasing Inappropriate Behavior by Ignoring and Redirecting; Decreasing Inappropriate Behavior – Following Through with Consequences; and Emotional Regulation, Social Skills, and Problem Solving. The IY TCM training is implemented using a group leader manual that promotes the integrity of the training through checklists, reminders, suggestions in the presentations and discussions in the workshops.

### Statistics

Pearson’s chi-squared test was used to compare the intervention and control conditions on gender and grade. A 2-level linear mixed models analysis with school as a level 2 random effect was used to compare the groups on baseline scores for SESBI-R, SSRS, STRS Conflict, STRS Closeness, TRF Internalizing problems, and TRF Academic.

Dropout at post-assessment was substantially higher in the intervention condition than in the control condition; this also applied to the group with severe externalizing problems (see flowchart). Hence, the data are not missing completely at random (MCAR) but may be missing at random (MAR). A mixed model was used with individuals as the random effect and the intervention and time (pre or post) as fixed factors. A mixed model includes all the available information at all time points in the analysis as well as individuals with data at only one time point. An alternative of using ANCOVA analysis (a regression analysis with the post score as the dependent variable and the pre score and condition as the covariate) would be based only on complete cases (complete case analysis) and would have been unbiased only if the data were MCAR. A mixed model, in contrast, is unbiased under the less restrictive MAR assumption and less biased than a complete case analysis if data are missing not at random (MNAR). A 3-level linear mixed model analysis was used to test for treatment effects. In this analysis, measurements (level 1) are nested within individuals (level 2) who in turn are nested within schools (level 3). Because we only have two measurements per individual, only the variance of random intercepts (and not slopes) at level 2 and 3 were estimated. Time and intervention group were treated as fixed factors, and the treatment effect was estimated as the effect of the time by group interaction in this model. All tests were evaluated using a two-sided .05 significance level. Effect sizes were computed as Hedges’ g. IBM SPSS Statistics (version 22) was used for all the analyses.

## Results

There were no significant differences between the intervention and control conditions at the baseline assessment in terms of grades, *χ* ^2^ (2, *N* = 83) = 0.33, *p* = 0.85, gender: *χ* ^2^ (1, *N* = 83) = 3.05, *p* = 0.81, or on scores on the measures, SESBI-R *t(33.7)* = −.52, *p* = 0.61, SSRS *t(58.6)* = −0.36, *p* = 0.72), STRS closeness *t(28.1)* = −0.35, *p* = 0.73), STRS conflict *t(28.5)* = −1.72, *p* = 0.10, TRF internalizing *t(27.5)* = 0.05, *p* = 0.96) and TRF academic *t(51.1)* = −0.48), *p* = 0.63). Table 2 shows baseline and follow-up data for the two conditions and the results of the mixed-model analyses with individuals as the random effect and the intervention and time (pre or post) as the fixed factors. There were statistically significant differences in the change from baseline to follow-up between the two conditions in teacher-reported student-teacher conflicts and academic performance. The intervention condition showed advantageous development in terms of student-teacher conflicts and improved academic performance. No statistically significant differences between the conditions were found in the change from baseline to follow-up in externalizing problems, social skills, internalizing problems and closeness with the teacher.

**Table 2:** Baseline and follow-up data for the two conditions, with results of the mixed model, *N* = 83

Assessment	Control condition		IY TCM condition		g	Effect of intervention		
	Baseline	Follow-up	Baseline	Follow-up		Estimate^a^	(95 % CI)	*p*-value
	Mean (SD)	Mean (SD)	Mean (SD)	Mean (SD)				
SESBI-R	168.58 (21.22)	158.05 (37.12)	171.22 (21.67)	148.48 (33.77)	−0.57	−11.87	(−27.4 to 3.68)	0.133
SSRS	66.04 (7.54)	67.86 (11.26)	66.74 (9.45)	71.10 (9.93)	0.29	3.12	(−1.09 to 7.32)	0.144
STRS:								
Conflict	20.42 (7.24)	21.37 (7.93)	23.06 (5.06)	20.00 (6.75)	−0.65	−3.83	(−6.80 to −0.86)	0.012
Closeness	27.40 (4.41)	27.27 (5.30)	27.78 (4.20)	28.51 (3.80)	0.20	1.34	(−0.56 to 3.24)	0.165
TRF:								
Internalizing	4.80 (6.89)	4.44 (6.85)	4.77 (4.79)	2.94 (3.36)	−0.25	−1.24	(−4.47 to 2.00)	0.448
Academic	2.73 (0.52)	2.69 (0.57)	2.81 (0.62)	3.06 (0.50)	0.50	0.24	(0.05 to 0.42)	0.014

Notes: Linear mixed model with time point (follow-up versus baseline), intervention condition (IY TCM), and their interaction as dichotomous covariates and individual as random effect. ^a^) Estimate and 95 % CI refers to the coefficient of the condition by time interaction. Observed sample size: Control condition Baseline, *N* = 37–38, Control condition Follow-Up, *N* = 35–36, IY TCM condition Baseline, *N* = 42–45, IY TCM condition Follow-up, *N* = 28–29. g = Hedges’ g

## Discussion

This quasi-experimental pre-post study evaluated the effectiveness of the Incredible Years Teacher Classroom Management Training program (IY-TCM) in a sample of 83 1^st^- to 3^rd^-grade Norwegian schoolchildren with severe externalizing problems. The IY-TCM was implemented in a naturalistic fashion that was comparable to any typical supplementary teacher training. Our hypothesis that there would be differences between the conditions—specifically, that the intervention condition would demonstrate a reduction in negative behaviors, improved social skills and fewer internalizing problems after the intervention—was not confirmed. We found no change in the students’ social and emotional problems after the IY-TCM intervention. This finding suggests that the effectiveness of the IY TCM as a stand-alone universal prevention program for Norwegian children who exhibit severe externalizing problems in school is not sufficiently comprehensive. This despite the fact that 41% of the children received special educational assistance. These children may be in need of a more specific, systematic and tailored intervention that is implemented over time [58] and is tailored to the needs and strengths of the child at home and in school [14]. An example to such an approach can be Response to Intervention (RTI), which is a multi-tier approach for early identification and support of children with behavior and learning needs [59]. The RTI process begins with high-quality instruction and a universal screening of all children in the regular classroom (Tier 1). Tier 2 refers to targeted interventions for children not making adequate progress in the regular classroom, and Tier 3 refers to more intensive interventions and comprehensive evaluations targeting a child’s skill deficits. Children in need of additional support are provided with interventions at increasing levels of intensity to accelerate their rate of learning. RTI can be a school-wide framework for efficiently allocating resources to improve student outcomes. If these children also exhibit externalizing problems at home, the simultaneous incorporation of parent training to strengthen parenting competencies might improve the outcome of the intervention [58, 60]. In a similar study of the effectiveness of the IY TCM conducted in elementary schools in the U.K., Hutchings [44] found that children with high scores in total difficulties showed a decrease in off-task behavior and negative behavior toward the teacher. One reason for the different results in the studies might be the use of different outcome measures.

Further, despite continued high levels of conflict, teachers in the intervention condition reported moderate change in student-teacher conflicts, whereas the control condition showed a tendency toward increased levels of conflict. Lower levels of conflict in relationships between teachers and students with severe externalizing problems after the IY TCM are a promising outcome. These students are at serious risk of negative student-teacher relationships [61, 62] because cascading impacts of negative student-teacher relationships on children’s externalizing problems have been found [63]. The quality of student-teacher relationships is one of the most influential elements within the learning environment and plays an important role in students’ functioning, both academically and socially [21, 62]. For students with severe externalizing problems, the quality of their relationship quality with their teachers has a significant effect on students’ academic engagement and achievement as well as their behavior, peer relationships and school adjustment [32, 62, 64]. A possible reason for this finding might be that the IY TCM provided the teachers with new skills and techniques to manage their students, thereby helping to reduce the teachers’ levels of stress, which allowed them to provide a more positive classroom environment for the students and contributed to better behavior.

A moderate change in improved academic performance was also found in the IY TCM condition. Previous research on interventions focusing on universal preventive efforts of externalizing problems suggested that students’ academic skills are affected more quickly than their mental health [65]. Our results support this finding by indicating that the academic skills of the children exhibiting severe externalizing problems were improved, whereas the mental health of these children was not significantly affected. Further studies across longer time spans are needed to investigate these processes. Nevertheless, this finding is especially important for children at risk, such as those who exhibit severe externalizing problems, since academic skills in the early elementary years are critical for later academic achievement [66] and future positions [67]. Students’ behavioral problems, such as inattention and disruptive behaviors, reduce their academic commitment, which frequently leads to reduced academic achievement [19]. A classroom that is managed better and fewer conflicts with the teacher may enhance students’ self-motivation for learning and have positive impacts on school achievement [21, 62]. However, this finding might be biased. Teachers’ perceptions and evaluations of students’ academic skills might be influenced by the presence of fewer conflicts rather than reflecting a change in academic performance.

Gottfredson and Gottfredson [68] found that the implementation quality of school-based prevention practices is often weak and that low-quality delivery of a program may not produce any results. Poor implementation quality may therefore explain our lack of findings. The time difference between the teacher's final workshop and the final assessment varied depending on the time of commencement. The final assessments were normally between 3 and 4 weeks after the teacher’s final workshop, and the short time span might have had an impact on the results. Despite few significant results in this study at the follow-up assessment, a similar Norwegian 4-year follow-up study reported more positive changes 3 years after implementation, with more evident changes in the intervention condition compared to the control condition [69]. It is unknown and impossible to predict how the behavior and social skills of the children in this sample will develop in the future; however, similar developments might occur.

### Limitations

This study has some limitations that require consideration. First, the study used a quasi-experimental design rather than a randomized control trial. Implementation of the IY TCM was dependent on available and qualified group leaders in the municipalities, making random assignments difficult. In addition, extensive predefined criteria for the implementation of the IY TCM recommended by IY Norway had to be fulfilled, which reduced and excluded a number of applications. Furthermore, information about the children’s behavior was based solely on teacher reports, reducing the quality of information. However, teacher ratings have been shown to be reliable [70], and similar results has been found in a Norwegian sample of adolescents [71]. Another limitation is the substantial dropout rate in the intervention condition, which may have influenced the results in the pre-post evaluations. A limited sample size also limited the power to detect differences between the two conditions. Additional limitations is that the implementation quality of the study was not measured, nor was the teacher’s willingness to implement the changes to their practice, in which can raise concerns about the effectiveness of the intervention. An additional limitation is the absence of blinding, which may have introduced bias into the results. Blinding of intervention and control conditions for the schools, teachers, students and parents was judged to be impossible in this study. Informing all schools of the nature, purpose and procedures of the research project evaluating the IY TCM program was necessary to gain agreement from the schools. Once schools were aware of their status in the evaluation, it was impossible to fully blind the teachers and students.

### Implications

One implication of this study may be that students in 1^st^ – 3^rd^ grade who exhibit severe externalizing problems require more comprehensive and tailored interventions than the IY-TCM intervention provided here as a universal prevention program. A diversity of interventions across different contexts, such as home and school, with equal goals for changes in the child’s negative behavior may enhance the effect and maintain it over the long term. Another option would be to tailor interventions to specific children beyond the universal intervention. The possible outcomes of these strategies are unknown, but for children who exhibit behavioral problems, the treatment modality is particularly important [58].

## Conclusion

Our findings show that the IY Teacher Classroom Management program is not sufficient as a stand-alone universal program in a Norwegian school setting for students with severe behavioral problems. We found no significant differences in the baseline to follow-up change in terms of disruptive behaviors, social functioning, internalizing problems or student-teacher closeness in the two conditions. However, some important secondary findings were found, including changes from baseline to follow-up in the IY TCM condition, a reduction in student-teacher conflicts and improved academic functioning. Nevertheless, young schoolchildren with severe behavioral problems require more comprehensive and tailored interventions.
